# The revelations of Q. Dissemination and resonance of the QAnon conspiracy theory among US Evangelical Christians and the role of the Covid-19 crisis

**DOI:** 10.1007/s41682-023-00147-2

**Published:** 2023-03-01

**Authors:** Heiko Beyer, Niklas Herrberg

**Affiliations:** Institute for the Social Sciences, Heinrich Heine University Düsseldorf, Düsseldorf, Germany

**Keywords:** Conspiracy theories, Evangelicalism, QAnon, Corona pandemic

## Abstract

Previous studies show that the QAnon conspiracy theory is especially popular among American evangelical Christians. The paper investigates the reasons behind this relationship. We hypothesize a mediation relationship between evangelical dogma and how it is practiced on the one hand and the susceptibility for conspiracy thinking on the other hand. We argue that evangelicalism due to its biblicism is characterized by the belief that its perception of reality holds absolute truth (nomization), that the world can be clearly divided into good and evil (Manichaeism), and that salvation can be achieved through political means (immanent eschatology). Those beliefs, in turn, in the uncertain times of the Covid crisis resonate with the cognitive (epistemic), the affective (moral), and conative (eschatological) elements of conspiracy theories. Using data of waves 46 (March 2019), 68 (April 2020), and 73 (September 2020) of the American Trends Panel, conducted by the PEW Research Center we show respective mediation effects: Evangelical Christians are particularly convinced that their religion alone holds absolute truth and that religion has not enough influence on politics. The latter also correlates with the conspiracy belief that “powerful people intentionally planned the Covid outbreak”. QAnon support again is linked both to such Covid related conspiracy thinking and the three elements of nomization, Manichaeism, and immanent eschatology.

## Introduction

In the course of the Covid-19 pandemic, the popularity of conspiracy theories has increased dramatically. For instance, a survey of US Americans conducted by the PEW Research Center (2020) found that around one in four of those polled can imagine that the Covid pandemic was intentionally planned. Obviously, the belief in the sinister power of unscrupulous groups has an appeal to many. As we will show, there are cognitive, affective as well as conative reasons for this.

Especially one conspiracy theory has become very popular during the pandemic and that is QAnon. Essentially, QAnon is a conglomerate of different beliefs, speculations, alleged evidence and explanations that center around the idea that a shadow government—known as the “deep state”—is controlling the destiny of the US, and that President Donald Trump and his administration’s main goal is to fight and destroy this cabal (see Rothschild 2021). By the time of the storming of the Capitol in Washington on January 6, 2021 QAnon left the digital space for good and demonstrated its potential for political mobilization (see Bond Bayleigh and Neville-Shepard 2021; Armaly et al. 2022).

Along with far-right groups such as the Proud Boys, it is especially evangelical Christians who support the QAnon conspiracy theory (Dinulescu 2021; MacMillen and Rush 2022). But the popularity of Donald Trump among evangelicals is not the only explanation for the situation (Margolis 2020). We suspect, rather, that there is a deeper ideological link between evangelical worldviews and conspiracy theory narratives.

Section 2 provides a brief introduction to the history and ideological characteristics of the QAnon movement. Against this backdrop, we will then explain in Sect. 3 to what extent QAnon can be considered an ideal type of conspiracy theory. Section 4 describes the general and specific features of US-American evangelicalism and discusses the extent to which conspiracy theories have resonated with its followers—particularly during the Covid crisis. Finally, the model developed is supported by data from the American Trends Panel of the Pew Research Center, thus empirically determining to what extent the support for QAnon among evangelical Christians is mediated by similar ideological motives and Covid-related conspiracy narratives.

## The QAnon conspiracy theory

Although conspiracy theories are far from a new phenomenon (van Prooijen and Douglas 2017), they have gained considerably in popularity over the past few years as a result of the rise of right-wing populism (van Prooijen et al. 2022; Castanho Silva et al. 2017) and protests against measures to contain the Covid pandemic (Bodner et al. 2020; Eberl et al. 2021). In a US-American context, the QAnon conspiracy theory in particular attracted a great deal of public attention—especially after the storming of the Capitol on January 6, 2021, in which key QAnon activists were proven to have been involved. While in February 2020 a total of 76% of Americans reported that they had never heard of QAnon, this figure was down to only 53% in September 2020, and a mere 39% claimed to be unaware of QAnon in March 2021.^1^ In the following step, we would like to briefly outline the origins and developments of QAnon. While describing the transformation of a fringe online phenomenon into a full-fledged social movement, we will point out the main elements of the underlying conspiracy theory narrative.

Already in 2016, during the election race between Hillary Clinton and Donald Trump, the increased importance of conspiracy theories was becoming apparent (Rothschild 2021; Bleakley 2021). Donald Trump’s campaign was very much focused on the subject of corruption of political elites in Washington, which was repeatedly portrayed as a “swamp” in need of draining. During this period, the concept of the “deep state” also gained in strategic importance for Trump and his base. Even after his election victory, Trump was able to continue to present himself as a political outsider and alternative to the establishment. The US President’s cryptic allusions to the “deep state” were taken up in online forums and imageboards, disseminated, and appropriated (Bleakley 2021).

In retrospect, we also have to interpret a comment by Donald Trump on the occasion of a state dinner with high-ranking US military personnel in the light of this, when he described the meeting as “the calm before the storm.” A few weeks later, one of the anonymous users (so called “anons”) on the imageboard 4chan was to claim that the “impending storm” was also related to the alleged imminent arrest of Hillary Clinton:

“Hillary Clinton will be arrested between 7:45 AM–8:30 AM EST on Monday—the morning on Oct 30, 2017.” (qalerts.app)

Taking up this claim, another user in the same thread on 4chan asked the following questions with reference to the state dinner:

“Why does Potus [sic] surround himself w/ generals? What is military intelligence? Why go around the 3 letter agencies?” (qalerts.app)

In the posts that followed, the user claimed to be a high-ranking official in the Trump administration and to have “Q clearance”—that is, comprehensive security authority within the US administration. He maintained this gave him access to hitherto confidential plans of the Trump administration (Rothschild 2021). Over the next few years, almost 5000 more posts from Q followed (known as “Q drops”), regularly informing the growing number of readers on the imageboards in cryptic codes about “the plan” and the allegedly successful strive of Trump and his staff. The content of these purported leaks from a source close to the White House ranged from discrediting prominent Trump critics and announcing state persecution of political opponents to smear campaigns against immigrants, liberals, and left-wingers, nationalist slogans, and antisemitic narratives, or completely unintelligible news about allegedly imminent events (Aliapoulios et al. 2021).

Since its inception in October 2017, some specific characteristics of the QAnon conspiracy theory have emerged. One important factor here is the correlation between the drops from Q and their interpretations. On the one hand, the vagueness and ambiguity^2^ of the “information” facilitates the exegesis of what Q “really” means. Supporters of the conspiracy theory themselves are invited to help expose the truth through their own interpretation of the Q drops—here, great importance is attached to those identified in QAnon jargon as “bakers,” who actively conduct an exegesis of the Q drops and thus also promote the dissemination of the conspiracy theories. At the same time, Q proclaims that the alleged strategy of Donald Trump and his administration is to be trusted implicitly and everyone should submit to this—“Trust the plan!” is one of the main slogans of the QAnon movement (Rothschild 2021; Aliapoulios et al. 2021).

Moreover, the Q drops, which are open to interpretation, allow the QAnon conspiracy theory to easily be linked to other conspiracy theory narratives and sentiments. In light of this, the QAnon theory entails not a single narrative, but a combination of different tropes and stories held together by a clear-cut Manichaeism: Trump and his supporters are fighting against evil in America and the world (Beverley 2020; Vrzal 2020). Particularly if we consider the images of the diabolical elite who drink children’s blood, control politics, the economy, and the media, and rule the world through socialism and feminism, the resemblance with antisemitic conspiracy theories, which of course are much older (Bergmann 2016), becomes clear. Furthermore, as well as these global classifications, events such as 9/11, the Kennedy assassination, or the outbreak of the Covid pandemic in China’s Wuhan are also interwoven into the interpretations, and these are explained with recourse to the plans and actions of those “Satanic elites.” Mike Rothschild (2021) therefore describes QAnon succinctly as a “Conspiracy Theory about everything.”

Moreover, in the context of the 2020 presidential campaign, it became clear that QAnon, albeit initially a form of online cult surrounding the messianic figure of Donald Trump, has increasingly gained prominence in the analog world, too (Demuru 2020). Far-right memes, images with quasi-religious iconography and easily identifiable symbols—in particular, the letter “Q” and the slogan “Where we go one, we go all (WWG1WGA)”—all of which originally emerged in remote online spaces became increasingly common at Trump’s rallies, demonstrations, and political events (Demuru 2020).

Especially in light of the Covid crisis and Joe Biden’s election victory, the QAnon movement continued to gain momentum and was able to mobilize its supporters in order to extend their activities to the analog world. Sustained by prophecies of a final struggle against the “cabal” of elites, QAnon developed into a scene prepared to use violence and to overthrow US democracy. The storming of the Capitol on January 6, 2021 has so far been the pinnacle of this radicalization (Bond and Neville-Shepard 2021).

## General characteristics of conspiracy theories

In the growing research literature on conspiracy theories a clear and universally accepted definition of the phenomenon is not in sight yet. In particular, the question of the veracity of a conspiracy theory, i.e. whether conspiracy theories must by definition be untrue, continues to be the subject of controversial discussions. There is agreement, however, that a conspiracy theory contains the assumption of an intentional and secretly acting (super-)powerful group (see Cubitt 1989), so that, as Barkun (2003) points out in his definition, coincidences do not exist and nothing is as it seems. These aspects can also be found in the definition of Baden and Sharon (2021), who emphasize that conspiracy theories “assume conspirators’ pervasive control over events and information” (Baden and Sharon 2021, p. 82). Furthermore, there is agreement that conspiracy theories entail a Manichean good-evil dualism, which can be found explicitly in Cubitt’s definition as well as in the work of Baden and Sharon and Douglas et al. (2019). Not necessarily an intrinsic element, but rather a more or less direct consequence of conspiracy theories, is their influence on behavior. They, for example, affect media consumption (Enders et al. 2021), voting behavior (Jolley et al. 2022) and the willingness to engage in political violence (Vegetti and Littvay 2021).

Taking these notions into account, we argue that QAnon can almost be considered an ideal type of a conspiracy theory. This assessment is supported by a review of the following three dimensions: (1) the epistemic/cognitive dimension, explaining the (social) world and thus reducing cognitive complexity (2) the moral/affective dimension, regarding the provision of a clear moral framework and the elimination of affective ambivalences and (3) the conative/action-related dimension, outlining a possible course of actions to bring about desired changes.

The epistemic function of conspiracy theories is to make sense of events taking place in the external world (Anton 2011). Through conspiracy narratives—in the case of QAnon, the conspiracy of satanic elites against American citizens—abstract political, social, and economic processes are personalized and causally explained (Douglas et al. 2019, 2016; Brotherton et al. 2013). The oldest and still most popular personalization today is the antisemitic one, according to which “the Jews pull the strings behind the scenes” and run the world. Even in cases where conspiracy theories do not directly refer to a “Jewish world conspiracy,” antisemitic codes and stereotypes are frequently used to characterize those allegedly responsible (on the mechanisms of antisemitism, see also Beyer 2015).

Apart from epistemic explanations, conspiracy theories also offer clear cut moral evaluation standards. Distinguishing themselves from “evil” conspirators and their corrupted followers allows conspiracy theorists to present and perceive themselves as people with integrity and sound morals. In essence, the underlying moral distinction consists of dualistic good-versus-evil semantics, which not only valorizes one’s own group and devalues the group of conspirators (Cubitt 1989; Butter 2018) but consistently dehumanizes the latter. For QAnon supporters, Hilary Clinton, Nancy Pelosi, and other members of the “deep state” personify an abstract diabolical principle.

Lastly, specific behavioral instructions are associated with this Manichaeism. The supposed recognition and associated evaluation of a conspiracy often leads to the conviction that “something has to be done” about the conspiracy. This might be an attempt to protect oneself against the conspiracy or to gather information about it. Since the end, sometimes the destruction of evil itself, is considered to justify all means, ultimately conspiracy theories have a considerable potential for violence (Amarasingam and Argentino 2020; Vegetti and Littvay 2021). In the case of QAnon, the conative dimension manifests itself not only in the purchase of products designed to protect against digital surveillance but also in participation in demonstrations, which with the storming of the Capitol in January 2021 even culminated in an attempted coup d’état. The fact that conspiracy theories can lead to violence against dissidents and even to attempted paramilitary coups shows just how virulent the problem has become in recent years (Bond and Neville-Shepard 2021; Armaly et al. 2022).

## The general and specific features of evangelical ideology

Even when examined on a superficial level, the QAnon movement is reminiscent of a religious sect—not only functionally but also in terms of its substance, for instance when bleak doomsday scenarios are evoked and demonic forces are to be combated. In this regard, Cohen (2022) also refers to QAnon as a “religious apocalyptic digital cult”. In general, the observation of a close connection between religion and QAnon is supported by the most recent research of the phenomenon. Juergensmeyer (2022), for example, argues that QAnon appeals to violent religious groups. Bond and Neville-Shepard suggest that Trump can be seen as a messianic figure in the QAnon faith. They consider Trumpism a “presidential eschatology”. Furthermore Smith (2022), MacMillen and Rush (2022) and Beauchamp (2022) also propose to understand QAnon in religious categories. The latter draws a connection between QAnon and evangelical Christianity in the USA, whereby the Corona pandemic in particular promoted support for QAnon in evangelical circles—a finding for which we can also find clear evidence in our empirical results presented later.

But before we turn to the empirical analysis of our data, we will explicate the affinity between QAnon and (Christian) religious beliefs more carefully. For that purpose we will, first, revisit the key characteristics of religious world views. We follow Beyer and Schnabel (2019) here, who have carved out three elements based on a review of previous works on religious worldviews: nomization, Manichaeism and eschatology. Those three elements capture our cognitive, affective and conative dimensions of religious worldviews. In a second step we further specify the form of this content in the evangelical dogma. This allows us to compare them with the specifications of conspiracy theories we made previously.

First, religious worldviews are attempts to *construct meaning*. For believers they fulfil an epistemic function because religion serves to process everyday experiences by making them fit into a classification system acquired through socialization. In other words, from the perspective of the sociology of knowledge, religious worldviews are “nomoi”—socially shared interpretative schemata that structure cognition and are responsible for the construction of reality (Berger and Luckmann 1991 [1966], p. 115f.). A stable nomos ensures that an interpretation of the world is maintained routinely and unquestioned in the background. Religious worldviews achieve this primarily by linking the world order described by the nomos to a cosmic order (Berger 1967, p. 35). In comparison to secular attempts at “cosmization,” particularly scientific explanations (Berger 1967, p. 36) that acknowledge the limits of human construction of reality, religion claims that “everything has its meaning”: “Religion implies that human order is projected into the totality of being.” (Berger 1967, p. 37).

Due to their comprehensive and absolute claim, many religious worldviews tend to include statements about implications of their teachings for everyday life (Glock and Stark 1968; Huber 2003; Beyer and Schnabel 2019). Besides the function of religious worldviews as an epistemic system for interpreting the world, they fulfill a further role by tying these systems to *normative, morally* charged rules (Pickel and Krüggeler 2013). As social norms they spill over into the secular. Generally speaking, the separation of religious and political spheres of influence is a relatively late product of the process of secularization and is consequently found to a much lesser extent among less “secularized” (in the sense of more privatized and de-politized) religious groups. The normative content of religion—especially in religious denominations that claim an absolute validity of their norms—is most clearly expressed in the concept of sin and the associated Manichean distinction between good and evil (Delumeau 1990; Granoff and Shinohara 2012). Good is identified with the divine, with light and redemption, whereas evil acts as a compromising, dark, and ominous counter-principle. Sin is the manifestation of evil in the subject and “temptation” its modus operandi in the world (Marshall 2010).

In addition to epistemic and normative functions, a conative element must also be included into our consideration. This aspect is closely connected to the eschatological convictions of the respective religion. The concept of sin is complemented by the state of redemption on the one hand and divine punishment on the other. By means of eschatological doomsday narratives, both individual actions and the history of humanity and the cosmos are interpreted in a meaningful way—particularly in Christianity (Taubes 2009 [1947]; Schwarz 2000; Moltmann 2004) and to some extent also in other world religions (Walls 2010). Normally, these are scenes of apocalyptic purification, describing the judging of the disbelievers (or the sinful) and the rewarding of the faithful (or the repentant). In the history of religion, there have been repeated attempts to actively bring about doomsday by intervening in the secular order—political theology calls this to “immanentize […] the eschaton” (Voegelin 1952, p. 120).

In the doctrine of American evangelicalism, which is the key focus in the present paper, these three analytical elements of religious worldviews—nomization, Manichaeism, and eschatology—are found in almost pure form. It should be noted, however, that the term “evangelicalism” is an umbrella term for various Protestant religious groups (including Premillennialism, Pentecostalism, Restorationism, Baptists, Pietists, and Lutherans), which, when we look at the specific characteristics, have very different views and practice very different rituals (Dayton 2001).

However, irrespective of internal sub-divisions within the evangelical church its common feature is that evangelicals interpret the biblical word to the letter. Evangelicalism is defined by strict biblicism (Soper 1994, pp. 38ff.). This means, on the one hand, that believers consider only their own religion to be true and leading to salvation. Consequently, the conversion of unbelievers is an integral part of religious activity. On the other hand, the concept of sin—and, along with this the concept of punishment—is of key importance. Through sin, human beings assumingly have been separated from God, and only through faith in Jesus Christ can this separation be overcome. Hence, the fundamental difference between evangelicalism and other forms of Christianity is ultimately rooted less in the content of the dogma than in the active form given to that content and the intensity with which it is practiced (Soper 1994, p. 38).

This formal ideological characteristic, we conjecture, makes evangelicals particularly susceptible to conspiracy theories in general and to QAnon conspiracy theory (Hypothesis 1) in particular. Stable structures of meaning that provide cognitive certainty (nomization), moral clarity (Manichaeism), and a political framework for religious action (immanent eschatology) are more pronounced among evangelicals (Hypotheses 2a–c). The associated beliefs, in turn, latch onto the epistemic-cognitive, the moral/affective, and the action-related elements of QAnon (Hypotheses 3a–c).

An initial study (Armaly et al. 2022) has already pointed out the relevance of QAnon in connection with Christian nationalism: “QAnon lore (…) is littered with eschatological ‘end times’ and Manichean imagery regarding a final war (called ‘the Storm’) between a corrupt, evil elite and a virtuous, good people. (…) Although less well developed in scholarly literature, initial evidence seems to indicate that Christian nationalism may be tied to support for the QAnon movement and related conspiracy theories. (…) (T)here is ample evidence in reportage from before, during, and after the Capitol riots to motivate an expectation that Christian nationalism played a part in this novel form of American political violence. Several of Trump’s most prominent Christian supporters framed his efforts to undermine the 2020 election results in religious terms.” (Armaly et al. 2022, p. 4f.) We intend to deepen our understanding of the mechanisms underlying this observation by focusing primarily on the group of evangelicals, who the study by Armaly et al. found to exhibit the strongest nationalism, as well as by evaluating additional empirical data that examine the links between evangelicalism and conspiracy thinking.

Moreover, we assume that these relations are influenced by the social upheavals caused by the Covid crisis. Sobol et al. (2022) have already shown that religiousness has an influence on Covid-related conspiracy beliefs. Łowicki et al. (2022) found that this regards “only the most dogmatic and fundamentalist type of religiousness”. We will investigate whether this correlation applies to evangelicals specifically.

**Fig. 1:**
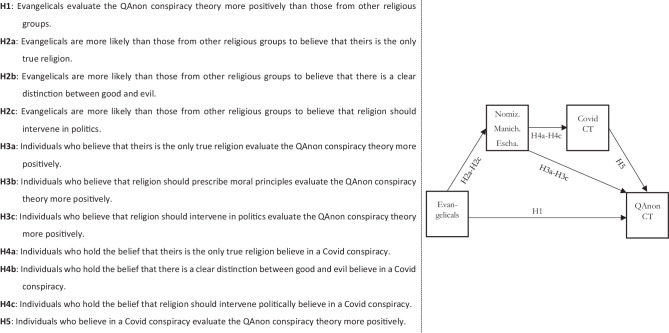
Hypotheses and model

Responding to heightened levels of cognitive, affective, and conative uncertainty, Covid-related conspiracy theories served as interpretive patterns, as emotional outlets, and as a way to turn passivity into activity (on the general connection between religious and political worldviews in times of social uncertainty, see Beyer and Schnabel 2019). We assume that evangelicals were more prevalent than other religious groups among supporters of Covid-related conspiracy theories. These specific conspiracy narratives were a gateway for other conspiracy theories—in the US context, primarily QAnon (Bodner et al. 2020)—because these theories are often connected through an underlying “conspiracy mentality” (Imhoff and Bruder 2014). Covid related conspiracy theories thus acted as a key mediator to establish the link between evangelical beliefs and QAnon (Hypotheses 4a–c and 5). We summarize the structure of our argument in Fig. 1 below.

## Method and empirical results

In order to examine the relationships outlined above, we draw on data from the American Trends Panel, conducted by the PEW Research Center, which surveys more than 10,000 Americans at regular intervals on changing political and social issues. We use panel waves 46 (March 2019), 68 (April 2020), and 73 (September 2020) for our analyses. By pooling these waves, we were able to accumulate information collected at different points in time, enabling a more valid operationalization of our theoretical concepts.

Since 2018, the sampling of the panel has been conducted using an address-based method in which households are first randomly drawn from the U.S. Postal Service Register (DSF). The household member is then selected using the last-birthday method. That person is asked to complete the questionnaire online, with individuals without internet access being provided with a tablet. The sample is representative of the US resident population aged 18 and over.

For our analyses, we used a subsample of individuals who reported belonging to a religious group and for whom responses to all variables in the final model (see Table 1 below) were available (*n* = 1240). We had to reduce the sample because the main mediator variables (nomization, Manichaeism, immanent eschatology) refer to religious beliefs and for that matter only vary for religious individuals. In total, 48.9% of this sample were female, 6.3% were in the 18–29 age group, while 27.7% were between 20 and 49, 33.5% between 50 and 64, and 32.4% aged 65 and over. Of these, 61.2% had at least a college degree, 26.0% had attended college without graduating, and 12.7% had a high school diploma or lower.

**Table 1:** Overview of the variables of the mediation model

Concept	Operationalization	n	m	Sd	Min	Max
QAnon support	“QAnon is a … somewhat good/very good thing for the country.” (W73)	1240	0.175	0.380	0	1
Covid conspiracy theory	“The theory that powerful people intentionally planned the Covid outbreak is … definitely true/probably true.” (W68)	1240	0.173	0.379	0	1
Evangelicals	“Would you describe yourself as a born-again or evangelical Christian, or not?” (W46)	1240	0.305	0.461	0	1
Nomization	“My religion is the one true faith leading to eternal life.” (W46)	1240	0.300	0.458	0	1
Manichaeism	“Churches and religious organizations … strengthen morality in society?” (W46)	1240	0.655	0.476	0	1
Immanent eschatology	“Churches and religious organizations have … not enough influence in politics.” (W46)	1240	0.247	0.431	0	1
Trump belief	How often, if ever, do you think each of the following get the facts right when it comes to the coronavirus outbreak? Donald Trump and his administration [at least “some of the time”] [W68]	1240	0.540	0.499	0	1

*Notes*: data from waves 46 (March 2019), 68 (April 2020), and 73 (September 2020) from the American Trend Panel of the PEW Research Center; unweighted data; listwise missing values; reduction on the basis of the model in Fig. 2

**Fig. 2:**
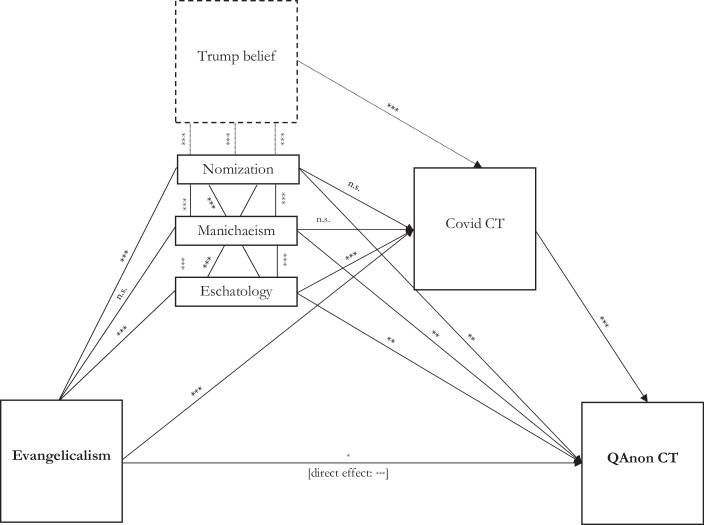
Linear probability models with mediation effects (bias-corrected percentile bootstrap); *n* = 1240; ***p < 0.001; **p < 0.01, *p < 0.05; all effects are positive effects

Support for QAnon was captured by asking whether the respondent felt QAnon was good or bad for the country (the US). Due to the non-normal distribution of the variable—an issue with all the measures used here—we recoded the original variable into a dummy variable: Those who indicated QAnon was “very good for the country” or “somewhat good for the country” were grouped into the “supporters” category and everyone else into the “opponents” category. A total of 17.5% (s = 0.38) of the subsample (*n* = 1240) of religious respondents expressed a positive view of QAnon (see Table 1). Significant differences between religious respondents and those who stated that they did not belong to any religious group could only be observed in comparison to Christians according to a simple (direct effect) linear probability model (LPM; see model M1 in Table 2).^3^ It is primarily Christians who support QAnon and more precisely evangelical Christians as can be seen if we look at the data for religious respondents only (see model M2 in Table 2).

**Table 2:** Direct effects linear probability models of QAnon and Covid conspiracy beliefs

	Model M1 (QAnon)	Model M2 (QAnon)	Model M3 (Covid consp.)	Model M4 (Covid consp.)
(Intercept)	0.08^***^	0.13^***^	0.13^***^	0.20^***^
	(0.01)	(0.01)	(0.01)	(0.01)
Christian	0.11^***^	–	0.12^***^	–
	(0.01)	–	(0.01)	–
Jewish	−0.03	−0.08^**^	−0.05	−0.12^***^
	(0.02)	(0.02)	(0.02)	(0.02)
Muslim	0.04	−0.01	0.01	−0.06
	(0.07)	(0.06)	(0.05)	(0.05)
Buddhist	0.04	−0.02	−0.04	−0.12
	(0.06)	(0.05)	(0.04)	(0.04)
Hindu	−0.04	−0.09	0.13^*^	0.06
	(0.04)	(0.04)	(0.07)	(0.07)
Other	0.02	−0.04	0.03	−0.05
	(0.03)	(0.03)	(0.03)	(0.03)
Evangelical	–	0.15^***^	–	0.12^***^
	–	(0.02)	–	(0.01)
R^2^	0.03	0.04	0.02	0.02
Adj. R^2^	0.02	0.04	0.02	0.02
Num. obs.	5125	3005	9597	6238

*Notes*: unstandardized coefficients; bootstrapped standard errors in parentheses; reference categories: not affiliated (M1/M3), other Christian denominations (M2/M4); ^***^p < 0.001; ^**^p < 0.01; ^*^p < 0.05

In total, 17.3% (s = 0.38) of our sample believe in the conspiracy theory that the Covid pandemic was deliberately planned (see Table 1)—more or less the same frequency we found for the QAnon support measure. Here again, it is Christians in particular who agree significantly more frequently than nonbelievers—but Hindus also expressed a similar view (see model M3 in Table 2). Among religious respondents, evangelicals again stand out, as suspected (see model M4 in Table 2).

At this point, a clear trend can be identified that corresponds to our theoretical conjectures: Evangelicals not only more frequently believe in the QAnon conspiracy theory (H1) but appear to be more susceptible to conspiracy theories in general. According to our line of argumentation, this can be explained by the form in which religious doctrine is practiced: Through the strict biblicism of evangelical Christianity, other religions are, on the one hand, generally denied their claim to truth and their interpretation of the world, and, on the other hand, evangelicals’ own moral normative code and corresponding concept of salvation are made absolute and politically charged.

In the studies conducted by the American Trends Panel, the item “My religion is the one true faith leading to eternal life.” lends itself to operationalizing the first point. A total of 30% (s = 0.46) of our subsample (*n* = 1240) agree with this statement. The belief that religion should interfere with politics in order to “immanentize the eschaton” (Voegelin 1952, p. 120) is reflected in the PEW item: “Churches and religious organizations have not enough influence in politics.” 24.7% (s = 0.43; *n* = 1240) of religious respondents agree with this statement. 65% (s = 0.48; *n* = 1240) believe that churches and religious organizations help strengthen the morals of society which we consider a measure for the importance of clear good-evil boundaries (Manichaeism).

We will now apply a mediation model (Hayes 2022) to test whether the causality structure between evangelical beliefs and belief in conspiracy theories can be confirmed as described above. For this, the PROCESS function of the R package “bruceR” (Bao 2022) was used to estimate a serial multiple mediation relationship between evangelical religious affiliation, religious beliefs, belief in Covid-related conspiracy theories, and support for QAnon. In order to control for evangelical’s above average support for Donald Trump, we also added a variable measuring if respondents believed Donald Trump’s statements about the Coronavirus.

The results of the mediation model can be found in Fig. 2 and in Table 3. First, a significant bivariate difference is also confirmed in the comparison between evangelical Christians on the one hand and all other religious groups (reference category) on the other hand: the probability that evangelicals consider the QAnon conspiracy theory being “good” or “very good” for the country (i.e., the US) is significantly higher. Thus, hypothesis 1 can be confirmed. In the mediation model the effect is still significant but the *p*-value much higher and the coefficient much lower—although the exact effect size should not be over-interpreted (see Fn. 3).

**Table 3:** Mediation linear probability model

	QAnon	Nomization	Manichaeism	Eschatology	Cov. Consp.	QAnon
(Intercept)	0.18^***^	0.00	−0.00	−0.00	0.17^***^	0.18^***^
	(0.01)	(0.01)	(0.01)	(0.01)	(0.01)	(0.01)
Evangelicalism	0.20^***^	0.38^***^	0.02	0.19^***^	0.11^***^	0.06^*^
	(0.02)	(0.030)	(0.03)	(0.03)	(0.03)	(0.03)
Manichaeism	–	0.11^***^	–	0.11^***^	−0.04	0.06^**^
	–	(0.02)	–	(0.02)	(0.02)	(0.02)
Eschatology	–	0.18^***^	0.15^***^	–	0.17^***^	0.08^**^
	–	(0.03)	(0.03)	–	(0.03)	(0.03)
TrumpCovid	–	0.09^***^	0.16^***^	0.15^***^	0.12^***^	–
	–	(0.02)	(0.03)	(0.02)	(0.02)	–
Nomization	–	–	0.14^***^	0.18^***^	0.04	0.07^**^
	–	–	(0.03)	(0.03)	(0.03)	(0.03)
Cov. Consp.	–	–	–	–	–	0.34^***^
	–	–	–	–	–	(0.03)
*R*^2^	0.06	0.30	0.11	0.21	0.13	0.21
Adj. *R*^2^	0.06	0.30	0.11	0.21	0.13	0.21
Num. obs.	1240	1240	1240	1240	1240	1240

*Notes*: unstandardized coefficients; standard errors in parentheses; ^***^p < 0.001; ^**^p < 0.01; ^*^p < 0.05

The weakening of the effect can be attributed to the correlation between evangelical affiliation and the ideological elements we introduced above. As we suspected there are significant mediation effects: Evangelical Christians believe more frequently than other religionists that theirs is the only true religion (nomization hypothesis H2a) and that religion does not have enough influence on politics (hypothesis on immanent eschatology H2c). Only the difference relating to moral certainty (Manichaeism hypothesis H2b) is not statistically significant. On the other hand, nomization and eschatology (as well as Manichaeism) have positive significant effects on how QAnon is evaluated (H3a–H3c). Further, beliefs in an immanent eschatology (i.e., that religion should be the guideline for politics) have an indirect effect by increasing receptiveness to Covid-related conspiracy theories (H4c) which again are strongly correlated with QAnon conspiracy beliefs (H5). The results are valid when controlling for the correlation between Trump support and evangelical ideology as well as belief in covid conspiracy narratives (all significant), respectively.

## Conclusion and Discussion

The aim of the present paper was to further conceptually and empirically specify the correlation between evangelicalism and support for the QAnon movement found in previous studies (Dinulescu 2021; MacMillen and Rush 2022; Bond and Neville-Shepard 2021; Amarasingam and Argentino 2020; Smith 2022; Cohen 2022). We focused especially on the impact of the Covid pandemic, adding to the more recent literature on the relationship between Covid-related conspiracies and religiosity (Łowicki et al. 2022; Beauchamp 2022). We were able to empirically back up our assumption that support for the QAnon conspiracy theory by evangelical Christians is mediated in particular by a functional equivalence between conspiracy theories and evangelical dogma as well as the conspiracy theory interpretations of the Covid pandemic.

Significant mediation effects clearly emerged with regard to the epistemic/cognitive and the conative/action-related dimensions. On the one hand, evangelical Christians are particularly convinced that their religion alone leads to salvation and that religion should influence politics. It is precisely this set of beliefs, on the other hand, that correlates with support of the QAnon movement. In the case of the conative dimension—religious influence on politics—the Covid pandemic functions as a catalyst: it shakes up behavioral routines and demands new legitimations for the state’s interference in people’s daily lives. Covid-related conspiracy theories provide the framework for a new cause of action by singling out responsible actors. They interpret the Covid pandemic as an intentional plot by political elites and call for an end to constraints on individual freedoms and a “return to normalcy”. Once the belief in a conspiracy is established, however, related narratives can be readily added. The relatively simple and case-specific Covid conspiracy theory thus became the gateway for further conspiracy narratives (in the US context: for QAnon in particular).

The result that particularly the immanent-eschatological element in evangelical dogma allows conspiracy theory narratives to resonate with its followers aligns with the evidence for the political impetus of other conspiracy theories (Douglas et al. 2019). Through its conviction that religion ought to have an influence on politics, evangelicalism has had a strong potential to mobilize its followers for the Covid and QAnon protests. Given this finding and that Bond and Neville-Shepard (2021) even characterize QAnon as a “presidential eschatology”, it would be instructive for future research to further specify the eschatological element in QAnon both theoretically and empirically.

Not all of our assumptions, however, were supported by the data. Evangelical Christians are not significantly more likely to believe in a Manichean struggle between good and evil—as measured by the consensus that churches and religious institutions improve social morality. We attribute this result less to a low relevance of Manichean beliefs in religious dogma and in conspiracy theory narratives, respectively, since those relationships have already been convincingly presented and empirically confirmed by many studies (Butter 2018; Cubitt 1989; Castanho Silva et al. 2017; MacMillen and Rush 2022). Rather, we assume that the empirical measure used here, in the absence of better alternatives, only partially reflects the underlying theoretical considerations. The item for Manichaeism taken from the American Trends Panel does not adequately address the good-versus-evil antagonism commonly referred to by the concept of Manichaeism. The reference to a strengthening of social morality by churches and religious institutions only implicitly addresses the belief in such an essentialist dichotomy (which again is connected to a strong belief in the notion of sin): to what extent “anti-religious” institutions (purposefully) undermine social morality is not directly explored. Consequently, future studies should use original scales and measures for this dimension of religiosity.

Another aspect that should be considered in subsequent analyses concerns the role of ideology for religious and conspiracy theory centered movements. As Armaly et al. (2022) argue with reference to the correlation between Christian nationalism and political violence, an analytical focus on (religious) ideologies often underestimates or even neglects the aspect of collective action and mobilization. Both, QAnon, having become a political force way beyond online image boards, and the evangelical movement in the US mobilize resources of different kinds—ideological ones being only one of the multiple tools which resource mobilization theory (McCarthy and Zald Mayer 1977) has introduced. The analysis of both movements’ connections to the media, for example, the role of Fox News in general and the role of individuals such as Alex Jones or Tucker Carlson in particular, or of politicians (primarily Donald Trump, of course, but QAnon believers elected to legislative bodies, too) as well as the roles of organizational elites, for instance, in the form of key evangelical preachers or QAnon multipliers on the internet, are thus important lacunas for future research.

## Appendix

**Table 4:** Mediation Analysis

Path	Coefficient	Bias corrected 95% CI
*Total Effect*	0.172	0.123, 0.222
*Direct Effect*	0.060	0.005, 0.127
*Indirect Effects*		
Evg → NOMIZ → QANON	0.028	0.005, 0.050
Evg → MANICH → QANON	0.004	0.001, 0.009
Evg → ESCHAT → QANON	0.018	0.003, 0.032
Evg → COVCON → QANON	0.037	0.017, 0.060
Evg → NOMIZ → MANICH → QANON	0.001	0.000, 0.003
Evg → NOMIZ → ESCHAT → QANON	0.003	0.001, 0.006
Evg → NOMIZ → COVCON → QANON	0.005	−0.003, 0.014
Evg → MANICH → ESCHAT → QANON	0.000	0.000, 0.001
Evg → MANICH → COVCON → QANON	0.001	−0.002, 0.000
Evg → ESCHAT → COVCON → QANON	0.014	0.008, 0.021
Evg → NOMIZ → MANICH → ESCHAT → QANON	0.000	0.000, 0.000
Evg → NOMIZ → MANICH → COVCON → QANON	−0.000	−0.001, 0.000
Evg → NOMIZ → ESCHAT → COVCON → QANON	0.002	0.001, 0.004
Evg → MANICH → ESCHAT → COVCON → QANON	0.000	0.000, 0.001
Evg → NOMIZ → MANICH → ESCHAT → COVCON → QANON	0.000	0.000, 0.000

*Notes*: Bias-Corrected Percentile Bootstrap Confidence Interval estimated based on 1000 Bootstrap samples

## Footnotes

1 Our own calculations based on data from the American Trends Panel of the PEW Research Centers (waves 62, 73, and 84; wording of the question: “How much do you know about QAnon?” Response category: “Nothing at all.”).

2 In this context, anthropologist Janet McIntosh pointed to the associated communication strategy of the “alt-right movement”: “Q-drops were examples of a broader communicative mode that has recently been evident among American members of the extreme right, or alt-right. Elsewhere, I have referred to this communicative mode as ‘alt-signalling’.” (McIntosh 2022) “Alt-signalling involves a mirroring of form and content, where indirect, ambiguous, or cryptic semiotic forms are used to allude to sinister, illicit, or conspiratorial dynamics. Before Q arrived on the scene, Donald Trump adopted a less dramatic precursor to this communicative style. He dog-whistled right and left, using coded statements that might sound benign to naïve listeners but excited white supremacists with thinly veiled racist allusions. Trump also tended to walk back his innuendo or controversial remarks as ‘just words’, ‘sarcasm’, or ‘joking’, leaving listeners uncertain about the relationship between sign and signified. And he used indirect and cryptic statements to stoke the idea that dastardly subterranean forces are at work among Democrats.” (McIntosh 2022, p. 9).

3 Note that since we are interested in the causal structure, the exact effect sizes are of lesser interest for us. LPM estimates are often biased due to issues of heteroskedasticity (although we are using bias-corrected percentile bootstrap techniques to account for this problem to some extent). But even the effect sizes of logit models with average marginal effects would be difficult to interpret so we opted to use LPM.
